# Behavioral stabilization through low-dose Clozapine in a youth justice center.

**DOI:** 10.1192/j.eurpsy.2025.1144

**Published:** 2025-08-26

**Authors:** C. Hidalgo Vazquez, Á. Armendáriz Lacasa, N. Del Prado Sánchez

**Affiliations:** 1 Consorci Sanitari de Terrassa; 2 Psychiatry, Parc Sanitari Sant Joan de Déu, Barcelona, Spain

## Abstract

**Introduction:**

Behavioral dysregulation and aggression pose substantial challenges in treating adolescents within youth justice systems, particularly in cases with complex psychiatric histories and diagnostic ambiguity. Autism spectrum disorder (ASD) is often underdiagnosed due to its different presentations, particularly in females. Many girls with ASD show strong desire for social interaction, but due to difficulties in navigating social relationships, they frequently experience frustration, resulting in explosive and auto and heteroaggressive behaviors. For those ASD patients with comorbid conduct disturbances, standard antipsychotic treatments can be insufficient, being necessary to try alternative pharmacological approaches.

**Objectives:**

To explore the therapeutic impact of low-dose clozapine on severe conduct dysregulation and auto/heteroaggression in this minor with ASD, and to assess clozapine’s effectiveness in reducing anxiety and improving adaptability within a structured therapeutic environment.

**Methods:**

We present a 17-year-old female patient with a history of severe behavioral disruptions with aggressiveness, initially diagnosed with various psychiatric conditions and treated with high-dose antipsychotics without clinical improvement. The case follows her through two separate admissions to a youth justice center’s therapeutic unit, where a retrospective review of her history and a longitudinal study of her diagnosis were made. After conducting a review of clozapine’s effects on reducing aggression, low doses of clozapine are finally tried, with the goal of stabilizing behavioral disturbances and reducing the patient’s associated distress.

**Results:**

When low-dose clozapine (up to 150 mg/day) was introduced, improvements in conduct regulation and emotional distress were documented. A treatment interruption occurred between admissions due to a lapse in its administration, followed by relapse in behavioral symptoms. Upon re-initiation of clozapine in the second admission, the patient’s symptoms stabilized again, with substantial behavioral improvement and reduced anxiety, enhancing the patient’s ability to function within the structured environment of the therapeutic unit. The patient achieved sustained clinical stability for over two months, a period marked by the absence of pharmacological rescue interventions or containment measures.

**Conclusions:**

Low-dose clozapine may be a viable option for managing severe behavioral disturbances in adolescents with ASD within youth justice settings, especially when other treatments prove ineffective. This case underlines the potential of clozapine not only to mitigate aggression but also to facilitate better adaptive functioning and reduce anxiety, contributing to a more stable therapeutic experience. Further investigation into clozapine’s role in treating conduct dysregulation in ASD is warranted.

**Disclosure of Interest:**

None Declared

